# P-312. Improving Utilization of PrEP Across Internal Medicine Resident Clinics

**DOI:** 10.1093/ofid/ofaf695.531

**Published:** 2026-01-11

**Authors:** Kaihei Masuda, Raakhi Menon, Migara Jayasekera, Alexander Bosley, Bisma Khwaja, Arkoon Ali, Anirudha Chatterjee, Christine Pho, Kumaraman Srivastava, Sanjana Janumpally, Joanie O’Leary, Muhammad Mushtaq, Michael Phan, Marysuna Wilkerson, Nadia Ahmed

**Affiliations:** The University of Texas Medical Branch, Galveston, Texas; The University of Texas Medical Branch, Galveston, Texas; The University of Texas Medical Branch, Galveston, Texas; The University of Texas Medical Branch, Galveston, Texas; The University of Texas Medical Branch, Galveston, Texas; The University of Texas Medical Branch, Galveston, Texas; The University of Texas Medical Branch, Galveston, Texas; The University of Texas Medical Branch, Galveston, Texas; The University of Texas Medical Branch, Galveston, Texas; The University of Texas Medical Branch, Galveston, Texas; The University of Texas Medical Branch, Galveston, Texas; The University of Texas Medical Branch, Galveston, Texas; The University of Texas Medical Branch, Galveston, Texas; The University of Texas Medical Branch, Galveston, Texas; The University of Texas Medical Branch, Galveston, Texas

## Abstract

**Background:**

The United States Preventive Services Task Force (USPSTF) recommends HIV screening for all adults and annual testing for those at higher risk, including men who have sex with men, individuals with STIs, injection drug users, and those engaging in transactional sex. Pre-exposure prophylaxis (PrEP), endorsed by the CDC, is a highly effective prevention method. Expanding PrEP education is crucial to reducing HIV transmission, improving health literacy, and addressing access disparities, especially among minorities and women. We aimed to increase HIV risk factor screening and appropriate PrEP prescription in the Internal Medicine resident clinic by 15% over a 3-month period.

**Image 1:**
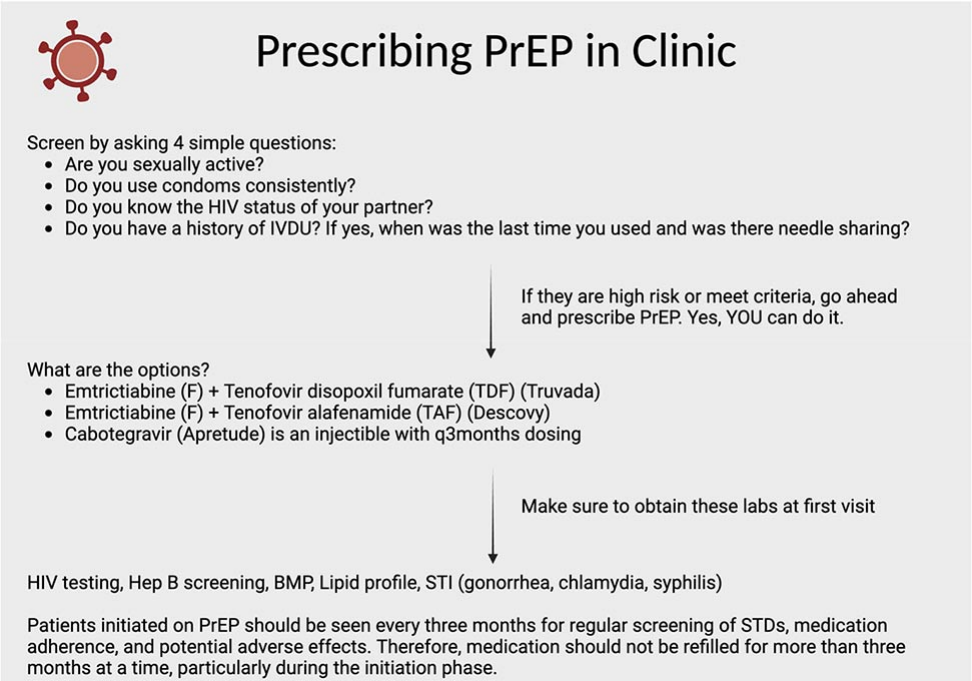
Educational posters in the clinic summarizing screening guidelines

**Figure 1:**
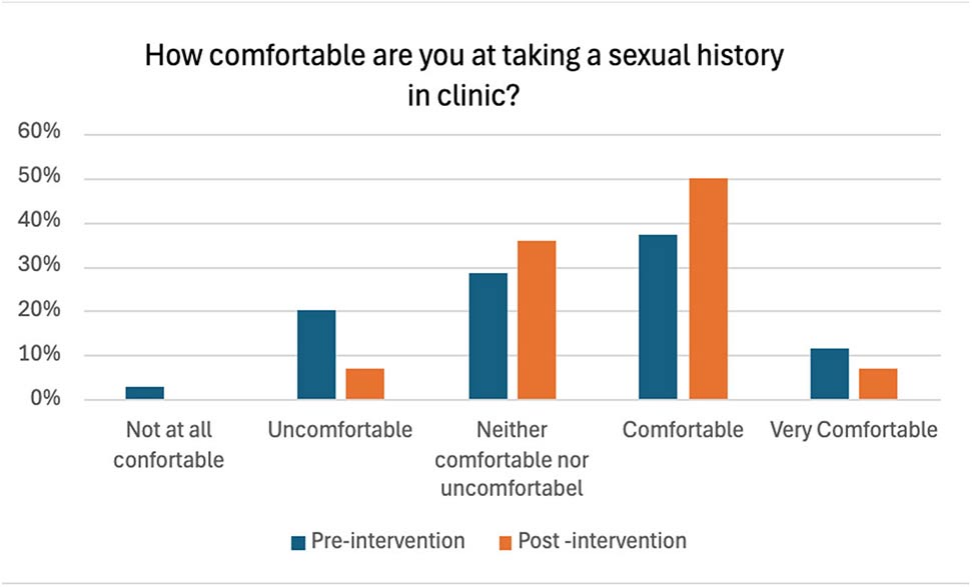
The pre- and post-intervention survey results (1)

**Methods:**

We reviewed 60 patient charts in the clinic to identify PrEP eligibility and current PrEP use. A pre-intervention survey assessed residents’ baseline knowledge, comfort with sexual history-taking, and experience prescribing PrEP. Based on survey results and feedback from residents, social workers, and infectious disease specialists, we developed targeted interventions: educational posters in the clinic summarizing screening guidelines, PrEP options, labs, and follow-up needs; and a self-directed PowerPoint module with a post-module questionnaire to assess knowledge gains.

**Figure 2:**
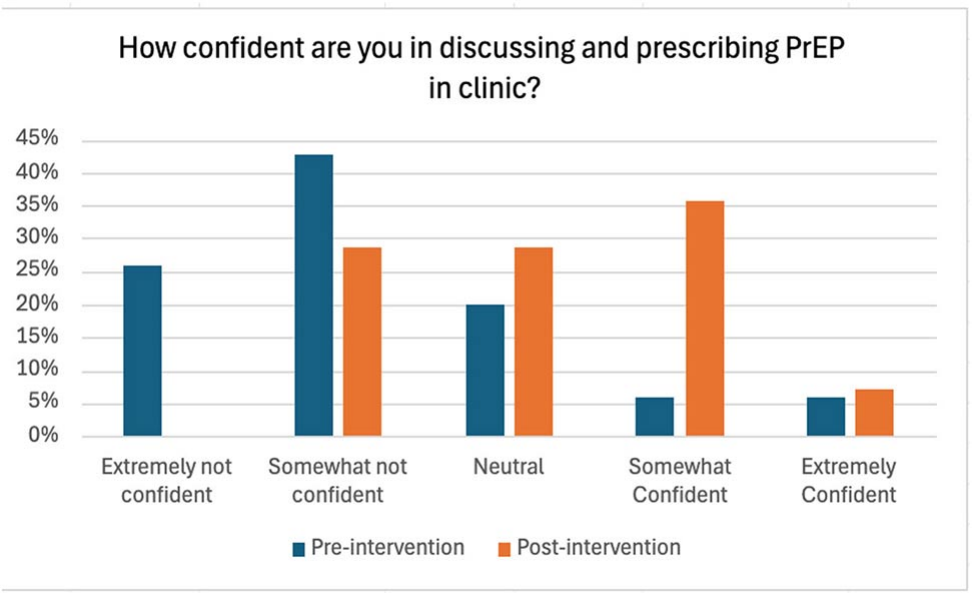
The pre- and post-intervention survey results (2)

**Figure 3:**
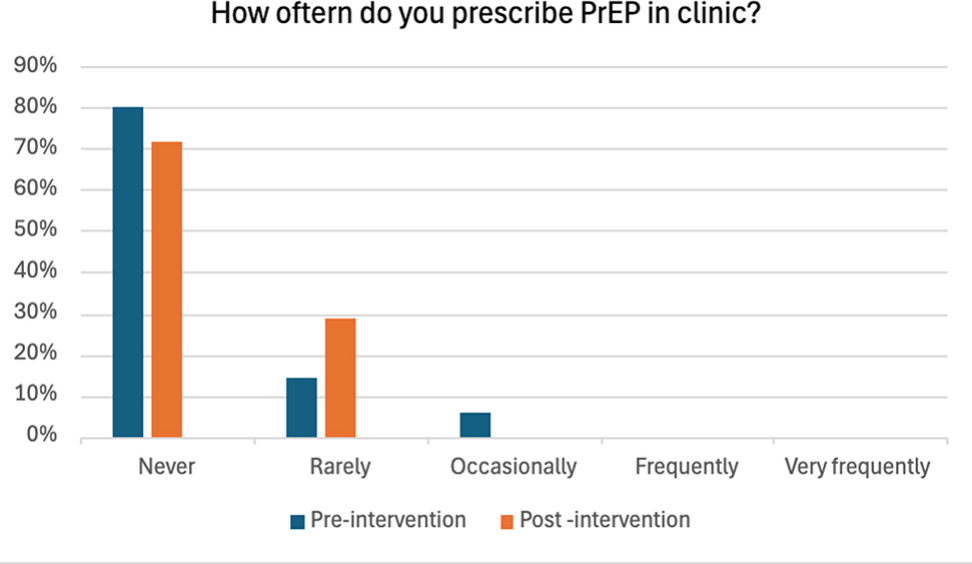
The pre- and post-intervention survey results (3)

**Results:**

At baseline, 33.3% of eligible patients were offered PrEP. The pre-intervention survey noted that 37% felt comfortable taking a sexual history, 80% had never prescribed PrEP, and 69% lacked confidence in discussing or prescribing it. After the intervention, the rate of PrEP offers to eligible patients remained at 33.3%. However, resident-reported comfort with sexual history-taking rose to 50%, and 28.6% reported prescribing PrEP. Confidence in discussing or prescribing PrEP improved, with only 28.6% of residents still reporting discomfort. Half of the residents also found the posters helpful in guiding conversations around sexual health and PrEP.

**Conclusion:**

There was not a significant increase in the rates of HIV PrEP prescription in the resident clinic over the course of a 3-month period. However, this project helped residents become comfortable discussing sexual health and PrEP in the clinic setting.

**Disclosures:**

All Authors: No reported disclosures

